# DF2726A, a new IL-8 signalling inhibitor, is able to counteract chemotherapy-induced neuropathic pain

**DOI:** 10.1038/s41598-019-48231-z

**Published:** 2019-08-13

**Authors:** Laura Brandolini, Vanessa Castelli, Andrea Aramini, Cristina Giorgio, Gianluca Bianchini, Roberto Russo, Carmen De Caro, Michele d’Angelo, Mariano Catanesi, Elisabetta Benedetti, Antonio Giordano, Annamaria Cimini, Marcello Allegretti

**Affiliations:** 1 Dompé Farmaceutici SpA, Via Campo di Pile, L’Aquila, Italy; 2 Department of Life, Health and Environmental Sciences, University of L’Aquila, L’Aquila, Italy; 3 Department of Pharmacy, University of Naples Federico II, Naples, Italy; 4 Department of Medical Biotechnology, University of Siena, Siena, Italy; 5 Sbarro Institute for Cancer Research and Molecular Medicine and Center for Biotechnology, Temple University, Philadelphia, USA

**Keywords:** Targeted therapies, Drug development

## Abstract

Chemotherapy-induced peripheral neuropathy (CIPN) is a common dose-limiting side effect of several anti-neoplastics and a main cause of sensory disturbances in cancer survivors, negatively impacting patients’ quality of life. Peripheral nerve degeneration or small fibre neuropathy is generally accepted as the underlying mechanism in the development of CIPN. Recent evidence has contributed to clarify the determinant role of cytokines and chemokines in the process leading to neuronal hyperexcitability. Exposure to oxaliplatin triggers alterations in peripheral neuropathic pathways previously linked to IL-8 pathway. We investigated a novel selective inhibitor of IL-8 receptors, DF2726A, and showed its effects in counteracting CINP pathways, extending the relevance of the activation of IL-8 pathway to the class of platinum chemotherapeutics. Based on our results, we suggest that DF2726A might be a promising candidate for clinical treatment of CIPN conditions due to its efficacy and optimized pharmacokinetic/pharmacodynamic profile.

## Introduction

Chemotherapy-induced peripheral neuropathy (CIPN) is side effect of different anti-cancer drugs resulting in sensory illness in cancer patients, affecting the patients quality of life^1,2^. Chemotherapeutic agents associated with neurotoxicity are taxanes, platinum drugs, vinca alkaloids, thalidomide and bortezomib^3^. To date, no therapy exhists for preventing and treating CIPN^1,4^. Therefore, a deeper understanding of the pathophysiology of CIPN to better identify new pharmacological targets and more specific treatments is mandatory.

CIPN affects approximately 70% of patients receiving chemotherapeutic treatments, and its development is directly related to the specific drug or combination of anti-cancer agents used, dosing regimen, and clinical conditions^2^.

Clinically, CIPN is characterized by a series of sensory symptoms including paraesthesia and dysesthesia manifested as numbness, tingling and altered touch perception as well as mechanical or thermal allodynia and hyperalgesia^5^. Even if the causes of CIPN are not fully clarified, it is noteworthy that the overall neuropathic symptoms are quite similar for all the chemotherapeutic drugs such as taxanes, platinum, proteasome inhibitors, and vinca alkaloids. Peripheral neuropathy is generally recognized as the main mechanism in the development of CIPN^6,7^, but different reports indicate that neuropathic pain caused by anti-cancer drugs may develop as early as the first treatment in the absence of to intra-epidermal nerve fibres (IENFs) damage or peripheric axonal degeneration^8,9^.

Recent evidence has contributed to elucidate the determinant role of cytokines and chemokines in the process leading to neuronal hyperexcitability. Exposure to chemotherapeutics such as paclitaxel^10^ and oxaliplatin^7^ consistently increased secretion of pro-inflammatory cytokines (TNFα, IL-1β and IL-6) and downregulated anti-inflammatory cytokines (IL-10 and IL-4) in spinal astrocytes and dorsal root ganglia (DRGs), triggering alterations in peripheral neuropathic pathways.

Paclitaxel was also associated with upregulation of Toll-like receptor 4 (TLR4) signalling, inducing higher expression of CCL2^11^ as well as recruitment of macrophages and pro-inflammatory T cells in DRGs^12^. In addition, preclinical *in vivo* results showed CCL2 overexpression in paclitaxel-induced spinal astrocytes and DRGs^13^. Similarly, a very recent report described the induction of CCL2/CCR2 pathway in oxaliplatin-induced DRGs, increasing mechanical and cold allodynia, and hypersensitivity of spinal astrocytes^14^. Several other studies found that both paclitaxel and oxaliplatin induced analogous mechanisms in the pathogenesis of neuropathic pain including spinal astrocyte activation, loss of IENFs, and mechanical and cold hypersensitivity^15–17^.

We recently reported the efficacy of reparixin, an inhibitor of CXCR1/2, in paclitaxel-induced CIPN in rats, identifying the activation of the IL-8/CXCR1/2 axis as a mechanism strictly implicated in the induction and maintenance of paclitaxel induced-neuropathy. In the present study, we investigated a novel selective inhibitor of IL-8 receptors, DF2726A, suitable for chronic oral administration, extending the relevance of the activation of IL-8 pathway to the class of platinum chemotherapeutics. Based on our results, we suggest that DF2726A might be a promising candidate for clinical treatment of CIPN conditions due to its efficacy and optimized pharmacokinetic/pharmacodynamic profile.

## Results

### DF2726A *in vitro* characterization

The main *in vitro* pharmacological and physicochemical results are summarized in Table 1.

**Table 1:** Physicochemical and *in Vitro* ADME Properties of DF2726A.

Assay	DF2726A
IC50	10 ± 5 nM (CXCL8) and 8 ± 3 nM (CXCL1)
Acqueous solubility	>30 mg/mL
pKa/logD_7.4_/logP	4.14/0.75/4.3
Plasma protein binding (10 µM) (human/dog/rat)	98.7%/94.8%/97.5%
Metabolic stability (human/dog/rat) (Test conc: 2.5 µM, µL/min/10^6^ cells	7.0/7.2/15.3
CYP Inhibition (IC50) (1A2/2C9/2C19/2D6/3A4)	>30 µM for all tested isoforms
CYP Induction (IC50) (CYP2B6, 1A2, 3 A)	>100 µM for all tested isoforms
Permeability (10 µM) (A to B/B to A/Ratio/pH 6.5/7.4)	27.8/14.6/0.52
hERG (IC50, patch clamp)	Negative (>1 mM)
AMES (100 µM)	Negative
COX 1-2 Inhibition (50 µM)	No Inhibition observed

*In vitro* pharmacological characterization showed that DF2726A did not inhibit spontaneous human polimorphonuclear neutrophil (hPMN) migration per se, but was equally efficacious in inhibiting IL-8- and CXCL1-induced hPMN chemotaxis with IC50 values calculated in 10 ± 5 nM and 8 ± 3 nM for IL-8 and CXCL1, respectively.

Counterscreens demonstrated >100-fold selectivity for 55 targets among GCPRs, ion channels, enzymes and transporters (see Supplementary Informations).

DF2726A displayed excellent physicochemical and *in vitro* ADME properties, with good aqueous solubility and low lipophilicity. High plasma protein binding in three species (human, dog and rat) was observed with values higher than 94%. Furthermore, DF2726A showed excellent stability toward liver microsomes in human, dog and rat, no inhibition of selected CYP-enzymes involved in drug-drug interactions and no CYP-induction. The permeability in the Caco-2 cell assay was good with no P-glycoprotein (P-gp) inhibition. DF2726A exhibited no hERG inhibition in a patch-clamp assay (IC50 > 1 mM) and was devoid of potential cardiovascular liability. The compound did not show any *in vitro* genotoxiticy as demonstrated in the AMES Test. Finally, DF2726A did not inhibit COX-1 and COX-2 when tested at 50 µM.

### Pharmacokinetic studies

Pharmacokinetic studies of DF2726A in Sprague Dawley rats showed a slow oral absorption (Cmax was reached at 6 h post dosing), high plasma exposure, a long half-life and low clearance (Table 2).

**Table 2:** Pharmacokinetic properties of DF2726A.

**Intravenous**	
t1/2 (h)	15.2 ± 3.8
AUC0-last (μg/mL·h)	140.8 ± 24.9
Vd (L/kg)	0.52 ± 0.09
CLtotal (mL/min/kg)	0.62 ± 0.11
**Oral**	
Cmax (µg/mL)	6.86 ± 1.6
tmax (h)	6.0
t1/2 (h)	16.2 ± 3.3
AUC0-last (μg/mL·h)	136.3 ± 22.6
F (%)	96.8

Data represent the mean of concentrations in rat plasma (n = 3) following a single 5 mg/kg intravenous dose and 5 mg/kg oral dose.

The oral elimination t_1/2_ of DF2726A was similar to intravenous elimination t_1/2_ (16.2 h vs 15.2 h). The relatively low volume of distribution for DF2726A can be explained by the high plasma protein binding observed in the rat (97.5%).

Interestingly, DF2726A showed a very high bioavailability (96.8%) and a suitable pharmacokinetic profile for oral treatment.

### DF2726A prevented oxaliplatin-induced peripheral neuropathy

**Figure 1:**
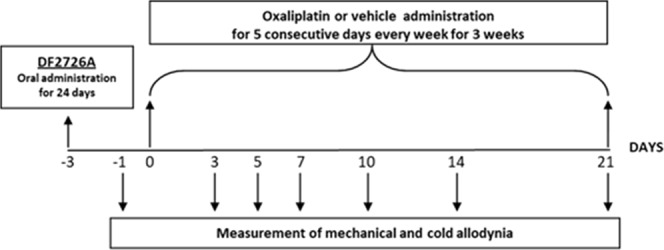
DF2726A and oxaliplatin administration schematic in rats.

Animals were treated according to the scheme illustrated in Fig. 1. Oxaliplatin-saline animals exhibited significative changes in paw withdrawal responses due to neurological toxicity.

**Figure 2:**
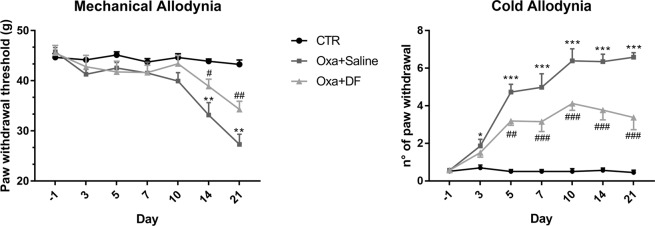
Time course of the effect of DF2726A on oxaliplatin-induced mechanical (**A**) and cold (**B**) allodynia. In control animals administered with oxaliplatin vehicle i.p. (CTR, black dots), the paw withdrawal responses remained unchanged during the whole experimental period. Rats treated with oxaliplatin (Oxa) + DF2726A vehicle (Oxa + saline, grey squares), showed marked changes in paw withdrawal responses. Administration of DF2726A (Oxa + DF, light grey triangles) was able to significantly reduce, oxaliplatin-evoked mechanical and cold allodynia. ***P < 0.001, **P < 0.01 and *P < 0.05 vs respective CTR group; ^###^P < 0.001, ^##^P < 0.01 and ^#^P < 0.05 vs the respective Oxa + Saline group. 2-way repeated-measures ANOVA and Bonferroni test. Data are expressed as mean ± SEM; n 10 per group. ANOVA, analysis of variance.

Specifically, in DPA test, the paw withdrawal threshold of oxaliplatin-saline-treated animals appeared strongly reduced only at 14 and 21 days (**P < 0.01) after the first oxaliplatin treatment. DF2726A showed a significant anti-allodynic effect at 14 (^#^P < 0.05) and 21 days (^##^P < 0.01) (Fig. 2A).

In cold allodynia experiments, no paw withdrawal response was induced by acetone in control animals, indicating that acetone-evoked cold stimulation is not noxious in non-neuropathic rats. Conversely, the paw withdrawal threshold was significantly increased at all experimental time-points in oxaliplatin-saline-treated animals (Fig. 2B). In particular, animals treated with oxaliplatin-saline showed a significant, persistent cold allodynia from day 3 (*P < 0.05) to day 21 (***P < 0.001) post-initial oxaliplatin dosing. DF2726A treatment produced a marked attenuation of cold allodynia starting from day 5 (^##^P < 0.01) and persisting until the final test day (^###^P < 0.001) (Fig. 2B).

The beneficial effect of DF2726A was not limited to the oxaliplatin-induced CIPN model; paclitaxel-induced mechanical and cold allodynia in rats was also prevented by administration of DF2726A (Supplementary Informations: Suppl Fig. 1).

**Figure 3:**
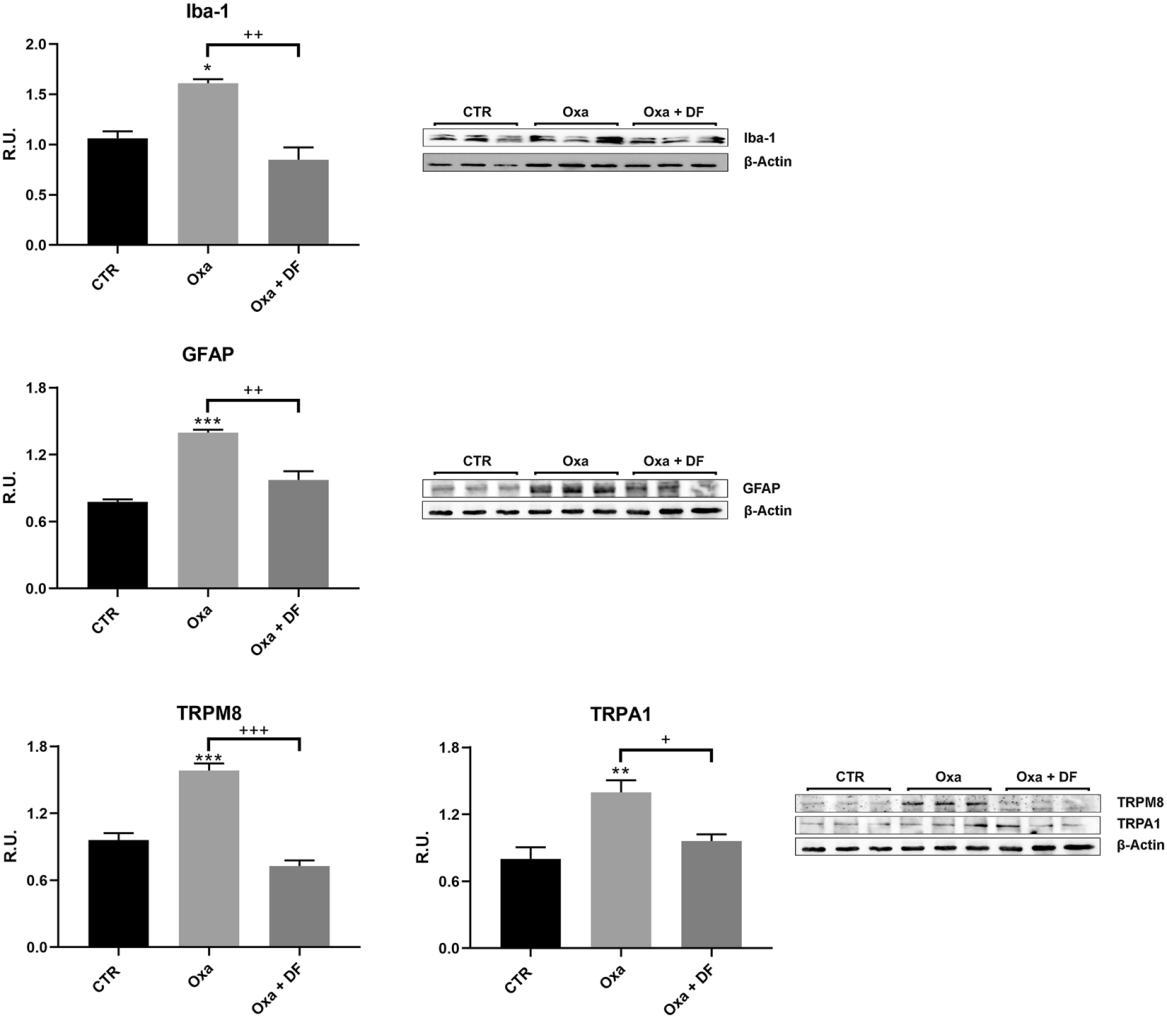
Western blotting analyisis for microglia marker Iba-1 and for astrocyte GFAP in control, OXA and (Oxa) + DF2726A rats. In the bottom, TRPA1 and TRPM8 receptors in in control, OXA and (Oxa) + DF2726A rats, evalutated by western blotting. Administration of DF2726A (Oxa + DF, light grey triangles) was able to significantly reduce reactive microglia and astrocytes as well as to reduce pain receptors. ***P < 0.001, **P < 0.01 and *P < 0.05 vs respective CTR group; ^+++^P < 0.001; ^++^P < 0.01 and ^+^P < 0.05 vs the respective Oxa + Saline group. 2-way repeated-measures ANOVA and Bonferroni test. Data are expressed as mean ± SEM; n 10 per group. ANOVA, analysis of variance.

Moreover, the activation of microglia and astrocytes in DRG was analysed through the expression of Iba-1 (Fig. 3) and GFAP (Fig. 3) by Western blot analysis. Results clearly showed a significant increase of both IBA-1 and GFAP expression in oxaliplatin rats compared to sham group (*p < 0.05 and ***p < 0.001, respectively). DF2726A treatment significantly reduced the expression of both these markers (^##^p < 0.01 vs OXA rats).

Finally, we also evaluated TRPM8 and TRPA1 expression in DRG. Also in these case both ionic receptor channels were up-regulated in oxaliplatin rats if compared to sham group (***p < 0.001) (Fig. 3). A significant reduction in the expression of both receptors was induced by DF2726A treatment (^#^p < 0.5, ^###^p < 0.001 vs CTR, respectively).

### DF2726A prevented oxaliplatin-induced loss of IENFs

**Figure 4:**
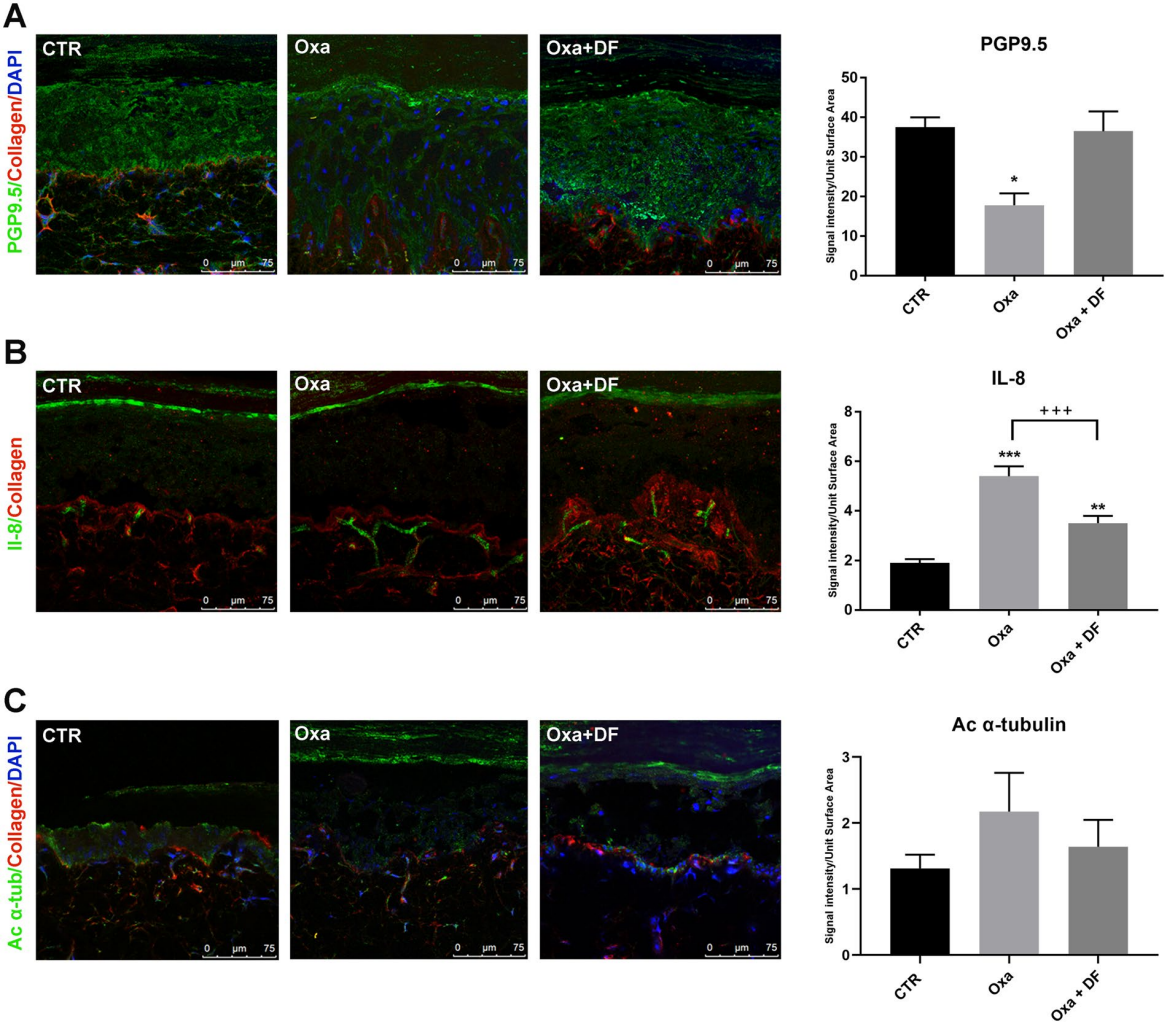
Effect of DF2726A treatment on oxaliplatin-induced intraepidermal nerve fibre loss. Representative images of immunofluorescence analysis and quantification of PGP9.5 (green, panel A), IL-8/CINC1 (green, panel B), acetylated a-tubulin (green, panel C) and collagen IV (red, panels A–C) in paw sections of rats from oxaliplatin vehicle group (CTR), oxaliplatin + DF2726A vehicle (Oxa + saline) and DF2726A-treated conditions (Oxa + DF). Nuclei are stained with DAPI (blue). **P < 0.005; ***P < 0.0005 vs CTR; ^+++^P < 0.0005 vs Oxa, unpaired t-test. Data are expressed as mean ± SEM. n 5 per group. Bar = 75 μm.

Loss of IENFs was reported to play a critical role in the development of various neuropathies occurring in response to chemotherapeutic agents, such as oxaliplatin^17^. The immunofluorescence assay of PGP9.5 (a marker of IENFs) and collagen IV showed damage in IENFs extending from derma into epidermis in oxaliplatin-saline-treated compared to control rats (Fig. 4A). Interestingly, DF2726A treatment strongly counteracted this effect, preventing the loss of epidermal innervation (Fig. 4A). In addition, immunofluorescence assay showed that oxaliplatin treatment increased IL-8/CINC1 signalling in the dermis. DF2726A treatment significantly attenuated this phenomenon, inducing a protective effect on nerve fibres (Fig. 4B). Lastly, anti-acetylated α-tubulin was evaluated in untreated, oxaliplatin- and DF2726A-treated nerve fibres. Immunofluorescence images showed that oxaliplatin treatment increased, even if not significantly, acetylated α-tubulin levels, while DF2726A completely restored control conditions (Fig. 4C). Taken together, these data show that oxaliplatin causes a pronounced decrease in IENFs, which was prevented by DF2726A administration.

### *In vitro* models

The viability assay in neuronal cells after different treatments showed that oxaliplatin and DF2726A were ineffective in modulating neuronal cell viability at a concentration range of 5–60 µM and 5–20 µM, respectively (Supplentary Informations: Suppl Fig. 2). For the subsequent experiments, DF2726A was used at a final concentration of 1 µM, and oxaliplatin at a final concentration of 20 µM. For comparison, paclitaxel alone or in combination with DF2726A was also used. Paclitaxel was ineffective in modulating cell viability in the concentration range 5–20 nM (Supplementary Informations: Suppl Fig. 2).

**Figure 5:**
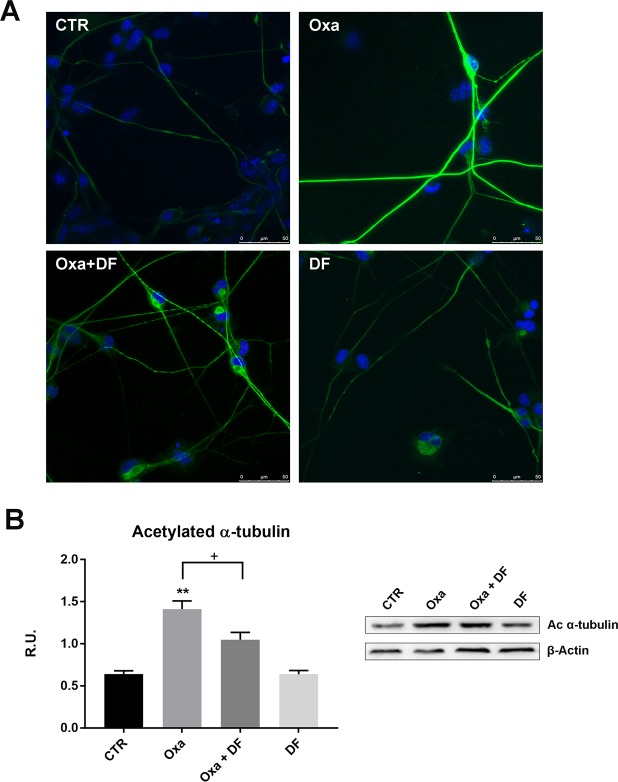
Effect of DF2726A treatment on acetylated α-tubulin in oxaliplatin-treated neurons. (**A**) Representative images of acetylated α-tubulin (green) in control (CTR), oxaliplatin- (Oxa), Oxaliplatin + DF2726A and DF2726A-treated neurons analysed at confocal microscope. Nuclei are stained with DAPI (blue). Bar = 75 μm. (**B**) Representative blots probed for acetylated α-tubulin (right panel) and densitometric analysis (left panel) of acetylated α-tubulin in the same treatment conditions. **P < 0.005 vs control; ^+^P < 0.05 vs Oxa, unpaired t-test. Data are expressed as mean ± SEM; n 3.

Acetylated α-tubulin (a marker of stable microtubules) was analysed by immunofluorescence in neurons after the different treatments. In untreated cells and in cells treated with DF2726A, acetylated α-tubulin was moderately present. In contrast, both oxaliplatin and paclitaxel -treated neurons showed an increase in acetylated α-tubulin levels (Fig. 5 and Supplementary Informations: Suppl Fig. 3). Specifically, fluorescence intensity in neurites was stronger, neurite diameter was greater, and cytoskeleton organization was more evident. Interestingly, acetylated α-tubulin in oxaliplatin + DF2726A-treated neurons was decreased compared to that in control conditions, indicating that the presence of DF2726A counteracts chemotherapy-mediated effects (Fig. 5). The same effect was observed for paclitaxel + DF2726A treatment (Supplementary Informations: Fig. S3). In addition, we observed a greater thickness in paclitaxel-treated neurites compared to control, while under combined treatment with DF2726A cells appeared as control cells (Additional file 5: Suppl Fig. 3).

Acetylated α-tubulin was also investigated by western blotting. In agreement with morphological data, both oxaliplatin and paclitaxel determined a marked increase in acetylated α-tubulin, while in combination with DF2726A the protein expression level was comparable to that of control cells (Fig. 5 and Suppl Informations: Suppl Fig. 3).

### DF2726A counteracted oxaliplatin-induced neurotoxic pathways

**Figure 6:**
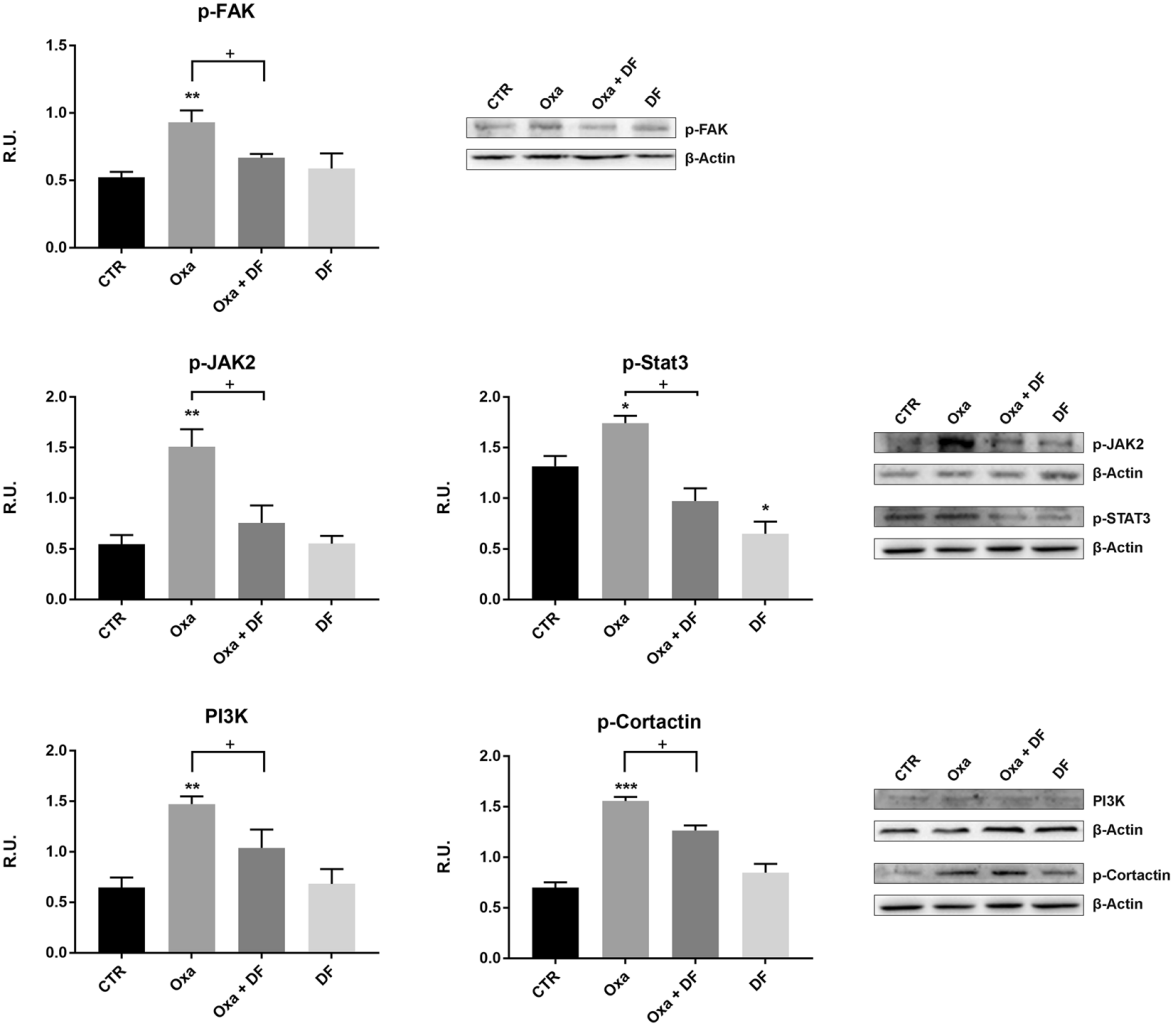
Effect of DF2726A treatment on oxaliplatin-induced neurotoxic signalling pathways in neurons. Representative blots of p-FAK, p-JAK2, p-STAT3, PI3K and p-cortactin and relative quantifications in control (CTR), oxaliplatin (Oxa)-, oxaliplatin + DF2726A (Oxa + DF)- and DF2726A (DF)-treated neurons. ***P < 0.0005; **P < 0.005; *P < 0.05 vs control; ^+^P < 0.05 vs Oxa, unpaired t-test. Data are expressed as mean ± SEM; n 3.

We investigated the effect of DF2726A on expression of the active form of the protein of focal adhesion (p-FAK), which is responsible for microtubule stabilization. p-FAK levels were significantly increased by both oxaliplatin and paclitaxel treatment, but decreased in the presence of DF2726A (Fig. 6 and Supplementary Informations: Suppl. Fig. 4). In addition, we analysed the active form of JAK2 (p-JAK2), involved in p-STAT3 signalling, which has a function in neuropathic pain and synaptic plasticity^18,19^. Both oxaliplatin and paclitaxel treatment increased p-JAK2, while combination with DF2726A restored control conditions (Fig. 6 and Supplementary Informations: Suppl. Fig. 4) p-STAT3 protein levels were also investigated in the different experimental conditions (Fig. 6 and Supplementary Informations: Suppl. Fig. 4). p-STAT3 levels increased after treatment with both chemotherapeutic agents compared to control, while DF2726A counteracted this effect (Fig. 6 and Supplementary Informations: Suppl. Fig. 4). We also investigated PI3K/p-cortactin pathway, which plays a role in axonal arborisation and synaptic plasticity. Both oxaliplatin and paclitaxel strongly upregulated PI3K/p-cortactin expression, while combination treatment with DF2726A attenuated this effect (Fig. 6 and Supplementary Informations: Suppl. Fig. 4). All together, these data suggest a common pathway activated by both taxane and platinum agents, and that DF2726A is able to counteract the effects of both drugs.

**Figure 7:**
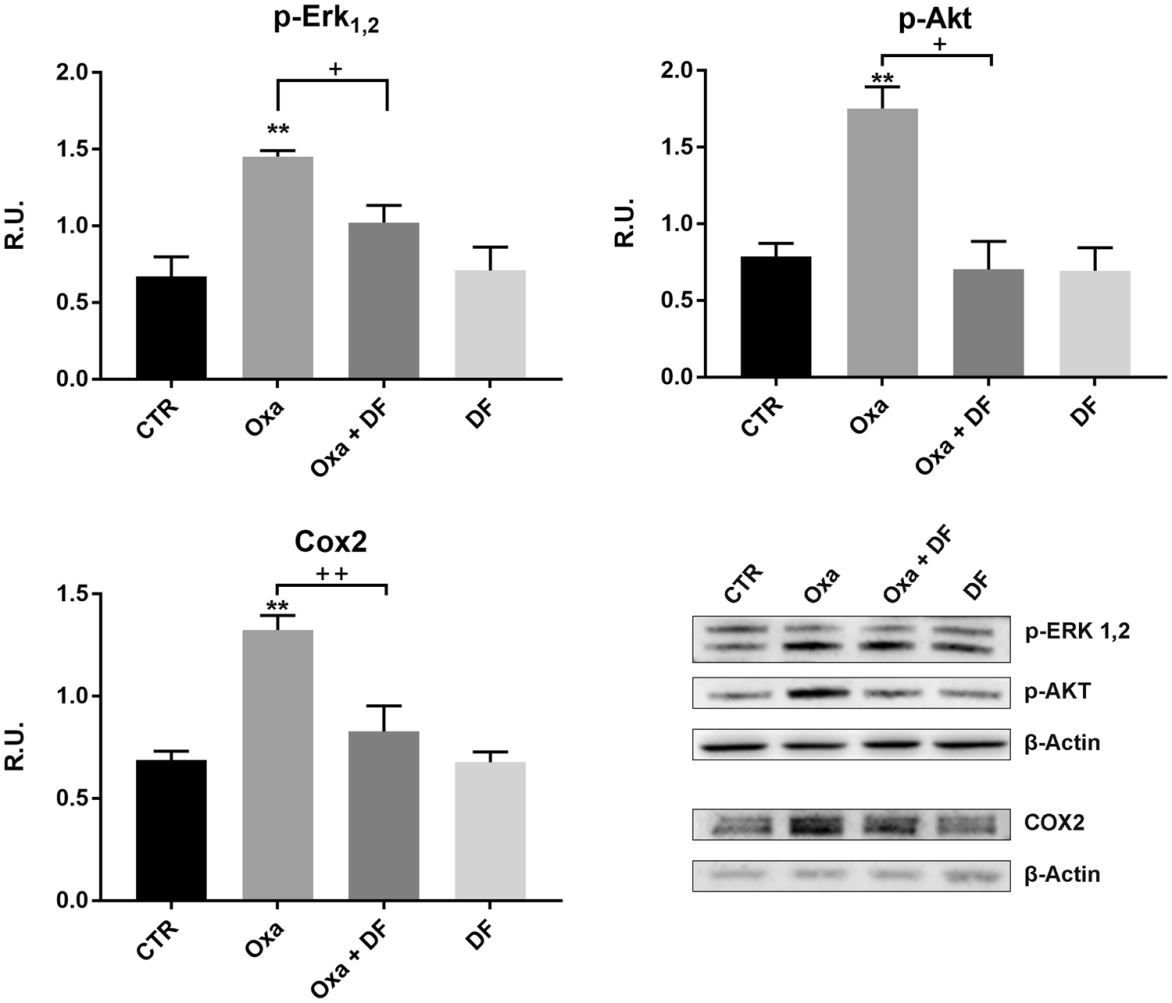
Effect of DF2726A treatment on p-ERK_1,2_, p-Akt and COX2 in oxaliplatin-treated neurons. Representative blots of p-ERK_1,2,_ p-Akt and COX2 and the relative quantifications in control (CTR), oxaliplatin (Oxa), oxaliplatin + DF2726A (Oxa + DF) and DF2726A (DF)-treated neurons. **P < 0.005 vs control; ^++^P < 0.005, ^+^P < 0.05 vs Oxa, unpaired t-test. Data are expressed as mean ± SEM; n 3.

Activation of MAPK pathways is known to contribute to cellular damage. Oxaliplatin activates p38 and ERK1/2 and promotes apoptosis in DRG neurons^20^. In our experimental conditions, oxaliplatin strongly upregulated the active form of ERK1/2 (p-ERK1/2) and increased PI3K and p-Akt, while in combination with DF2726A protein levels were comparable to those of control cells (Fig. 7). Lastly, oxaliplatin robustly upregulated COX2 protein levels, while in combination with DF2726A COX2 expression levels were comparable to the control (Fig. 7).

## Discussion

CIPN is a common adverse effect and the main dose-limiting toxicity associated with selected chemotherapeutic agents, including paclitaxel and oxaliplatin^1^. Although major efforts are now focusing on preventing the development of taxane- and platinum-induced acute and delayed CIPN without impacting clinical outcomes, no drugs have yet been approved for prophylaxis and/or treatment of CIPN. Current therapeutic strategies are based on the continuous modification of chemotherapy regimens to manage the symptoms of devastating side effects and to improve patients’ quality of life.

Platinum compounds bind to nuclear DNA of cancer cells, leading to aberrant re-entry into cell cycle, apoptosis and tumour degradation. Paclitaxel and oxaliplatin are the two chemotherapeutics most frequently associated with the development of severe untreatable CIPN. Oxaliplatin, a third-generation platinum chemotherapeutic agent, causes severe acute and chronic peripheral neuropathies, including cold allodynia, loss of sensitivity and motor disturbances^1,7,14^. The pathophysiological mechanisms of paclitaxel- and oxaliplatin-induced neuropathy are not completely understood. However, a large body of evidence describes an associative and causal relationship between activation of the immune system following taxane and platinum therapy and development of CIPN symptoms^10,12^. Several preclinical studies demonstrated that DRGs and spinal cord astrocytes treated with paclitaxel and oxaliplatin display higher levels of pro-inflammatory chemokines and cytokines (CCL2, CCL3, TNF-α, IL-6, IL1β and IL-8), as well as decreased anti-inflammatory cytokine expression (IL-10 and IL-4), causing sensitization of nociceptors, mechanical hyperalgesia and loss of IENFs^16,17^. A recent controlled clinical trial found an association between higher serum levels of pro-inflammatory cytokines and the severity of neuropathy induced by oxaliplatin in patients with colon cancer^21^. However, the exact mechanisms linking systemic cytokine and chemokine levels following chemotherapy with the development of CIPN symptoms remain to be elucidated.

Consistent with the above studies, our previous findings showed the significant effect of reparixin in attenuating the development of CIPN in sensitive neurons, suggesting a potential therapeutic role for chemokine inhibitors in prophylaxis and treatment of paclitaxel-induced CIPN^22^. We contributed to clarify the role of paclitaxel-induced IL-8 expression as a trigger of the progressive neural toxicity mediated by activation of the pathways responsible for microtubule stabilization, axonal arborisation and synaptic plasticity.

In the present study, we evaluated the efficacy of DF2726A, a novel optimized selective dual inhibitor of CXCR1 and CXCR2, in an oxaliplatin-induced neuropathic pain model, confirming the key role of these pharmacological targets and the additional importance of pharmacokinetic/pharmacodynamic optimization. Importantly, this work also extended our mechanistic and pharmacological studies of the paclitaxel-induced model, showing that the mechanisms underlying the neurotoxicity of the two drugs, unrelated to their chemotherapeutic action, are largely conserved and imply a key involvement of IL-8 receptors.

As previously reported for reparixin^22^, we found that DF2726A acts as a non-competitive allosteric modulator of CXCR1/2. DF2726A is a potent dual inhibitor of CXCR1/2 (IC50), with a good cross-reactivity versus rat and mouse, and an optimal selectivity profile versus a large panel of unrelated receptors. In line with the objectives of the lead optimization program, DF2726A exhibited improved features compared to reparixin, such as a longer half-life (16.2 h vs 1.78 h p.o. administration), a reduced plasma protein binding and better oral bioavailability. The optimization of the pharmacokinetic/pharmacodynamic profile was found to be associated with greater *in vivo* efficacy of DF2726A compared to that previously reported for reparixin in counteracting the increased sensitivity to tactile and cold stimuli induced by oxaliplatin and paclitaxel. The results reported here describe for the first time the efficacy of CXCR1/2 inhibition in *in vivo* models of oxaliplatin-induced peripheral neuropathy. DF2726A administration in rats afforded an attenuated level of behavioural hypersensitivity, suggesting that IL-8 signalling contributes to both taxane- and platinum-induced CIPN. In line with the *in vivo* effects, we observed that oxaliplatin led to a strong decrease in the number of IENFs, which was significantly counteracted by DF2726A. Although the exact mechanism of epidermal denervation is poorly understood, some studies suggested that it is linked to both paclitaxel- and oxaliplatin-induced neurological toxicity^17,23^, and is associated with increased levels of multiple pro-inflammatory cytokines^10,14,23^. Interestingly, we showed that IENF loss corresponds to a local increase in IL-8 induced by oxaliplatin, and that DF2726A treatment strongly attenuated this phenomenon, suggesting its important protective role in nerve fibres.

In order to dissect the signal transduction pathways underlying the function of DF2726A in alleviating both paclitaxel- and oxaliplatin-induced neuropathic pain, we assessed the effects of IL-8 signalling in DRG-derived neuron cultures. In cancer, alterations in microtubule stability, including acetylation of tubulin, are reported to influence cellular responses to chemotherapeutics, tumour development, cellular trafficking and survival^22,24,25^. Although not classified as anti-microtubule agents, platinum compound agents are also associated with the alteration of assembly processes and signalling functions of tubulin, as well as changes in cytoskeletal and axonal functions, all of which lead to neurotoxicity^3,25–27^. It was previously reported that DRG neurons express IL-8 receptor^22^. We investigated the effect of DF2726A inhibition on both taxane- and platinum-induced tubulin acetylation in DRG-derived neurons. In our experimental conditions, both paclitaxel and oxaliplatin determined an increase in α-acetylated tubulin in DRGs, an effect efficiently counteracted by exposure to DF2726A. Under both paclitaxel and oxaliplatin treatment, DRG-derived neurons expressed higher levels of p-FAK responsible for microtubule stabilization, and activated the PI3K/p-cortactin pathway, leading to terminal axonal arborisation and synaptic plasticity. Additionally, paclitaxel and oxaliplatin exposure increased expression of p-JAK2, thereby triggering p-STAT3, known to have a function in neuropathic pain and synaptic plasticity. In line with our previous findings^22^, we demonstrated that CXCR1/2 inhibition by DF2726A is effective in reducing p-STAT3, p-FAK and PI3K/p-cortactin activation induced by both taxane and platinum agents, by modulating pivotal markers of chemotherapy-associated neurotoxicity. The activation of COX2 and ERK1/2 pathways can further contribute to cellular damage and neuropathic pain^20,28,29^. Oxaliplatin treatment was reported to induce peripheral neuropathy by triggering the COX2 and p-ERK1/2 cascade pathways^20,28,30^. Notably, our study corroborated these findings and showed that DF2726A significantly reduces COX2 and p-ERK1/2 protein expression by counteracting their oxaliplatin-induced activation, thus suggesting a pivotal role for CXCR1/2 in activating the COX2/ERK cascade.

Our findings show that IL-8 and its receptors CXCR1/2 are critically involved in the development of neurotoxic activity associated with both paclitaxel and oxaliplatin treatment in DRGs, and CXCL8 blocking by DF2726A significantly counteracts their effects *in vitro* and *in vivo*.

Taken together, the results of our mechanistic studies suggest that IL-8 expressed in peripheral neurons may trigger a neuro-inflammatory reaction, resulting in a progressive neural sensitization via activation of key pathways involved in microtubule stabilization, terminal axonal arborisation and synaptic plasticity, and that the progressive accumulation of IL-8 may be partially responsible for the progressive epidermal denervation associated with chemotherapeutic treatments.

In summary, we show for the first time in a model of both paclitaxel- and oxaliplatin-related CIPN that the activation of IL-8 signalling is involved in the development of peripheral neuropathies, and that interfering with this signal using the novel compound DF2726A may be a useful strategy to alleviate chemotherapy-induced neurotoxicity.

## Methods

For the *in vitro* pharmacological-physicochemical characterization and pharmacokinetic studies of DF2726A see Supplementary Informations.

### Animals

Behavioural tests were performed on male Wistar rats (200–250 g, Harlan, Italy) housed in the animal care facility at the Department of Pharmacy of the University of Naples Federico II, Italy, as previously described^22^. Animals were housed, in a group of five, in a room with controlled temperature (22 ± 1 °C), humidity (60 ± 10%) and light (12 h per day); food and water were available *ad libitum*. All animals were weighted on the day of each treatment. All behavioural tests were performed between 09:00 and 17:00 h, and the animals were used only once. Animal care and manipulations were conducted in conformity with International and National law and policies (EU Directive 2010/63/EU for animal experiments, ARRIVE guidelines, and the Basel declaration including the 3R concept). The procedures reported here were approved by the Institutional Committee on the Ethics of Animal Experiments (CVS) of the University of Naples Federico II and by Ministero della Salute under protocol no. 2014-00884607. Rats were randomized and divided into equal-size groups (n = 10 group) not predetermined by a statistical method. After completion of experiments, animals were sacrificed by cervical dislocation.

### Drug administration

DF2726A was dissolved in saline and administered at the dose of 30 mg/2 ml/kg/os for 24 consecutive days starting 3 days before oxaliplatin administration and continuing for a further 21 days after the first administration of oxaliplatin. During this period, the compound was given 2 h after oxaliplatin treatment. For the drug administration regimen with paclitaxel, see Additional file 2.

### Induction of neuropathy by oxaliplatin

Rats received i.p. injections of oxaliplatin (Tocris) (2.4 mg/kg/day) or vehicle (5% glucose, 0.5 mL/rat), administered for 5 consecutive days every week for 3 weeks as described^22^.

Behavioural testing was performed prior to oxaliplatin/vehicle administration (day -1), as described in^22^ in order to determine the basal values of mechanical and cold nociceptive thresholds, and again on 3, 5, 7, 10, 14 and 21 days following oxaliplatin/vehicle injection as shown in Fig. 1. For induction of neuropathy induced by paclitaxel, see Supplementary Informations.

### Mechanical allodynia

To assess for changes in sensation or in the development of mechanical allodynia, sensitivity to tactile stimulation was measured using the Dynamic Plantar Aesthesiometer (DPA, Ugo Basile, Italy), which is an automated version of the von Frey hair assessment^22^. Animals were individually placed in Plexiglas boxes (30 × 30 × 25 cm) with a mesh metal floor covered by a plastic dome that enabled the animal to walk freely, but not to jump. When a trial is initiated, the device raises the filament to touch the foot and progressively increases force until the animal withdraws its foot, or until it reaches a maximum of 50 g of force (cut-off). The DPA automatically records the force at which the foot is withdrawn. This test does not require any special pre-training, but just an acclimation period to the environment and testing procedure. Each paw was tested twice per session and the test was performed on both paws on the day before (day -1) and then 3, 5, 7, 10, 14 and 21 days after first administration of oxaliplatin or vehicle. No consistent left and right differences were observed. The means of paw withdrawal (expressed in grams) were calculated from an average of four separate measurements. For assessment of mechanical allodynia using paclitaxel, see Additional File 2.

### Cold allodynia

Cold sensitivity was measured as the number of foot withdrawal responses after application of acetone to the dorsal surface of the paw^8,22^. Animals were individually placed in Plexiglas boxes (30 × 30 × 25 cm). A drop of acetone (25 μL) was applied to the dorsal surface of paws with a syringe connected to a thin polyethylene tube while the rats were standing on a metal mesh. A brisk foot withdrawal response, after the spread of acetone over the dorsal surface of the paw, was considered as a sign of cold allodynia. The procedure was repeated three times at 5 min intervals on both paws. The mean of paw withdrawal (expressed in numbers) was determined from an average of six separate measurements. Cold responses were measured before (day -1) and then 3, 5, 7, 10, 14 and 21 days after first administration of oxaliplatin or vehicle. No consistent left and right differences were observed. For assessment of cold allodynia using paclitaxel, see Additional File 2.

### Immunohistochemistry

Animals were sacrificed and a biopsy (3 mm) was taken from pad of the right hind paw. Biopsies were immediately placed in Zamboni’s fixative, where they were left at 4 °C. Tissues were then transferred to 20% sucrose for at least 24 h and used for immunohistochemistry analysis. Specimens were frozen in Optimal Cutting Temperature compound (OCT, Sakura Finetek Inc, CA, USA) and sliced into 20 μm thick serial coronal sections by cryostat AMES LAB-TEK (Westmont, Illinois, USA).

In brief, sections were blocked with phosphate buffer saline (PBS) containing 10% (Bovine Serum Albumin) and 0.3% Tween-20, for 1 h at RT and incubated, overnight at 4 °C, with the primary antibodies: IL-8 (mouse, 1:25, R&D Systems Inc. Minneapolis, MN, USA), PGP9.5 (rabbit, 1:1000, AbD Serotec, NC, USA), acetylated alpha tubulin (mouse, 1:250, Abcam Cambridge, MA, USA) and collagen IV (goat, 1:25, Southern Biotech, Birmingham, AL, USA). Sections were then rinsed in PBS several times before incubation for 2 h at RT with secondary antibodies, donkey AlexaFluor 488 anti-rabbit or donkey AlexaFluor 633 (1:2000), goat anti-mouse conjugated with Alexafluor 488 (1:2000; Life Technologies, Camarillo, CA, USA). After extensive washing, coverslips were mounted with Vectashield mounting medium with DAPI (Vector Laboratories Burlingame, CA, USA) and then observed at a Leica TCS SP5 confocal microscope (Leica, Mannheim, Germany)^31^.

### Quantification

Five slices were examined for each animal. For each slide, 4 fields were counted. For quantitative evaluation of IL-8/CINC-1 (the homolog of IL-8 in rat) and PDG95, photomicrographs for each condition were analysed by ImageJ software (National Institutes of Health, Bethesda, MD, USA) as previously described^31^. Briefly, the pictures were converted into grey scale, and arbitrary units were assigned: 0 = black (i.e. absence of signal) and 255 = white. The signal was first measured on the tissue in an area devoid of signal at visual inspection and assumed as background; the threshold was then set at 1.5 times the background and the surface area and mean grey intensity were measured for all areas above the threshold. To obtain the signal intensity (in arbitrary units), the background was subtracted from the mean grey intensity and the result was multiplied by the surface area above threshold. This value was divided by the surface area of tissue section to calculate the signal intensity per unit surface area.

### Cell culture and drug treatments

F11 hybridoma cells (ECACC 08062601), chosen as a model of DRG neurons^22,32,33^ were cultivated in DMEM (Euroclone, Milan, Italy) supplemented with 10% FBS (Sigma-Aldrich, St. Louis, MO, USA), 1% penicillin/streptomycin (Euroclone, MI, Italy) and 1% glutamine (Euroclone) at 37 °C, in humidified 95% air with 5% CO_2_ atmosphere. For all the experiments, cells were used at 18th passage. For immunofluorescence analysis, cells were seeded on coverslips at 1 × 10^4^ cells/cm^2^ for 24 h. Cells were then differentiated with rat NGF (rNGF; Sigma-Aldrich). rNGF was dissolved in DMEM with 1% penicillin/streptomycin and 1% glutamine (FBS free) at a final concentration of 50 ng/ml. Medium was replaced every 3 days until complete differentiation, which occurred after 7 days.

Following neuronal differentiation, neurons were treated for 24 h with DF2726A (1 μM final concentration), oxaliplatin (Sigma-Aldrich; 20 µM final concentration), or the combination of the two molecules.

DF2726A stock solution (4.3 mM) was prepared freshly by dissolving 1.5 mg of DF2726A in 1 ml PBS, 20 µl NaOH 1 N and 30 µl HEPES 1 M.

Oxaliplatin stock solution (40 mM) was prepared by dissolving the powder in DMSO, and aliquots were stored at -20 °C. For cell treatment with paclitaxel, see Additional File 2.

### Immunofluorescence

Cells were fixed in 4% paraformaldehyde in PBS for 20 min at RT and permeabilized in methanol for 5 min at −20 °C. Cells were then blocked with PBS containing 4% BSA for 30 min and incubated with the following primary antibody diluted in the blocking solution overnight at 4 °C: rabbit acetylated α-tubulin (1:4000; Cell Signaling Technology, Inc., Danvers, MA, USA), as previously described^22,33^. Cells were then rinsed in PBS several times before incubation with secondary antibodies: goat anti-rabbit conjugated with Alexafluor 633 (1:2000; Life Technologies, CA, USA) for 30 min at RT. After extensive washing, coverslips were mounted with Vectashield mounting medium (Vector Laboratories Burlingame, CA, USA) with DAPI and then observed at a Leica TCS SP5 confocal microscope (Leica).

### Western blotting

Animals were sacrificed and dorsal root ganglia (DRG) (L4-L6) were removed and stored at 80 °C. They were homogenized on ice-cold lysis buffer [20 mM Tris–HCl (pH 7.5), 10 mM NaF, 150 mM NaCl, 1% Nonidet P-40, 1 mM phenylmethylsulfonyl fluoride, 1 mM Na3VO4, leupeptin and trypsin inhibitor 10 μg/mL; 20 µl/sample, as previously described^34^.

After 1 h, tissue lysates were obtained by centrifugation at 20,000 g for 15 min at 4 °C. Protein concentrations were estimated by the Bio-Rad protein assay using bovine serum albumin as standard.

DRG lysate proteins (30 μg) were dissolved in Laemmli sample buffer, boiled for 5 min, and separated on SDS-polyacrylamide gel elctrophoresis and transferred onto nitrocellulose membrane (240 mA for 40 min at room temperature). The filter was then blocked with 1 × PBS and 3% non-fat dried milk for 40 min at room temperature and probed with anti-Glial Fibrillary Acidic Protein (GFAP) antibody (diluition 1.1000; cat.no.Z0334, Dako), anti-ionized calcium-binding adapter molecule 1 (Iba-1) antibody (dilution 1:1000; cat. no.019-19741, Wako), anti-transient receptor potential ankyrin 1 (TRPA1) antibody (diluition 1:1000; cat. no. NB110-40763, Novus Biologicals) and anti- Transient receptor potential M8 (TRPM8) antibody (diluition 1:10100; cat. no. NBP1-97311, Novus Biologicals) in 1 × PBS, 3% non-fat dried milk, and 0.1% Tween 20 at 4 °C overnight. The secondary antibody was incubated for 1 h at room temperature. Subsequently, the blot was extensively washed with PBS, developed using enhanced chemiluminescence detection reagents (Amersham Pharmacia Biotech, Piscataway, NJ, USA) according to the manufacturer’s instructions, and the immune complex visualized by Image Quant (GE Healtcare, Milan, Italy). The protein bands were scanned and densitometrically analyzed with a model GS-700 imaging densitometer (Bio-Rad Laboratories, Milan, Italy). To ascertain that blots were loaded with equal amounts of protein lysates, they were also incubated in the presence of the antibody against the β-actin protein (Sigma-Aldrich, Milan, Italy).

Control and treated cells were collected and lysed in ice-cold RIPA buffer (PBS pH 7.4 containing 0.5% sodium deoxycolate, 1% Igepal, 0.1% SDS, 5 mM EDTA, 1% protease and phosphatase inhibitor cocktails; Sigma-Aldrich) as described in^22,33^. Protein lysates (30 μg) were separated on 8–12% SDS-polyacrilamide gel and electroblotted onto polyvinyldifluoride membrane (PVDF; Sigma-Aldrich). Nonspecific binding sites were blocked by 5% non-fat dry milk (Bio-Rad Laboratories, Hercules, CA, USA) in Tris buffered saline (TBS: 20 mM Tris- HCl, pH 7.4, containing 150 mM NaCl) for 30 min at RT. Membranes were then incubated overnight at 4 °C with the following primary antibodies, diluted with TBS containing 0.1% Tween 20 (TBS-T) and 5% non-fat dry milk: rabbit acetylated α-tubulin (1:3000; Cell Signaling Technology), rabbit p-FAK (1:500; Santa Cruz Biotechnology, Santa Cruz, CA, USA), rabbit p-JAK2 (1:500; Santa Cruz Biotechnology), rabbit PI3K (1:500; Abcam, Cambridge, UK), rabbit p-cortactin (1:1000; Abcam), goat p-STAT3 (1:200; Santa Cruz Biotechnology), rabbit p-Akt (1:1000; Immunological Sciences, Rome, Italy), goat COX2 (1:500; Santa Cruz Biotechnology), p-ERK1/2 (1:500 Santa Cruz Biotechnology). As secondary antibodies, peroxidase-conjugated anti-rabbit or anti-mouse IgG (1:10000; Vector Laboratories) and anti-goat (1:1000; Santa Cruz Biotechnology) were used. Immunoreactive bands were visualized by Pierce ECL Substrate (ThermoFisher Scientific, Waltham, MA, USA), according to the manufacturer’s instructions. The relative densities of immunoreactive bands were determined and normalized HRP-conjugated β-actin, using ImageJ software. Values were given as relative units (RU).

### Statistics

For the *in vivo* results, all data were presented as mean ± SEM. Data analysis was performed using GraphPad Prism (GraphPad Software Inc., San Diego, CA, USA). The significance of differences between groups was determined by two-way analysis of variance (ANOVA) followed by Bonferroni post hoc tests for multiple comparisons. The level of significance was set at P < 0.05.

For the *in vitro* and *ex vivo* results (rat paw), data were expressed as mean ± SEM. Statistical analysis was performed by Student’s unpaired t test. The level of significance was set at P < 0.05.

### Ethics approval and consent to participate

The procedures reported for animal use were approved by the Institutional Committee on the Ethics of Animal Experiments (CVS) of the University of Naples Federico II and by Ministero della Salute under protocol no. 2014-00884607.

## Supplementary information

supplementary informations

## Data Availability

The datasets used and/or analysed during the current study are available from the corresponding author on reasonable request.
