# Supplementary material for: DF2726A, a new IL-8 signalling inhibitor, is able to counteract chemotherapy-induced neuropathic pain
Source: Sci Rep. 2019 Aug 13;9:11729. doi: 10.1038/s41598-019-48231-z (PMC6692352; doi:10.1038/s41598-019-48231-z)

Supplementary Information for "DF2726A, a new IL-8 signalling inhibitor, is able to counteract chemotherapy-induced neuropathic pain"

Authors: Laura Brandolini, Vanessa Castelli, Andrea Aramini, Cristina Giorgio, Gianluca Bianchini, Roberto Russo, Carmen De Caro, Michele d'Angelo, Mariano Catanesi, Elisabetta Benedetti, Antonio Giordano, Annamaria Cimini, Marcello Allegretti.

Materials

Chemokines were from PeproTech (London, UK). Chemicals and cell culture reagents were from Sigma Aldrich (Saint Louis, MI, USA).

Cell isolation and culture

Human mononuclear cells and PMNs were obtained from buffy coats of heparinized peripheral blood from adult healthy volunteers courtesy of Centro Trasfusionale, Ospedale S. Salvatore, L'Aquila, Italy. Ethical clearance was obtained to perform these experiments. Human PMNs were prepared to 99% purity by dextran sedimentation followed by hypotonic lysis of contaminating red blood cells as previously described [1]. PMNs were washed once with saline and then resuspended at 1.5 x 10^6/ml in HBSS for migration assay. Cell viability, as measured by Trypan Blue dye exclusion, was greater than 98%.

Migration assay

Migration of human PMNs was evaluated by microchamber technique in a 48-well microchemotaxis chamber, as previously described [2]. Briefly, 25 µl of control medium (PBS for monocytes and HBSS for PMNs) or chemoattractant (CXCL1, CXCL8) solutions were seeded in the lower compartment of the chemotaxis chamber. Fifty µl of cell suspension (1.5 x 10^6/ml) pre-

incubated at 37°C for 15 min in the presence or absence of different concentrations of DF2726A or vehicle were seeded in the upper compartment. The two compartments were separated by 5 µm pre-size polycarbonate filter (polyvinylpyrrolidone-free for PMN chemotaxis). The chamber was incubated at 37°C in air with 5% CO_2 for 45 min (PMNs), or 2 h (monocytes). At the end of incubation, filters containing migrated cells were removed, fixed, stained with Diff-Quik and 5 oil immersion fields at high magnification (100X; Zeiss microscope) were counted after sample coding.

Physicochemical characterization

The main physicochemical properties of the compound (pKa, $logD_{7.4}$, logP and solubility) were determined using the SiriusT3 apparatus (Sirius Analytical Instruments Ltd., East Sussex, UK) equipped with an Ag/AgCl double junction reference pH electrode, a Sirius D-PAS spectrometer and a turbidity-sensing device. The titration experiments were conducted in 0.15 M KCl solution under argon atmosphere at a temperature of 25 ± 1°C.

Selectivity

DF2726A was tested at Eurofins Cerep SA (France) by radioligand binding assays to assess the off-target activities towards a panel of GPCRs, enzymes, ion channels, transporters and nuclear receptors. All the selected targets are recommended by four major pharmaceutical companies [3]. DF2726A was dissolved in DMSO to achieve 10 mM stock solution, which was diluted with water/HBSS to a final concentration of 10 µM. Cell membrane homogenates (48 µg protein) were incubated for 60 min at 22°C with the respective reference compound in the absence or presence of the test compound in a buffer containing 50 mM Tris-HCl (pH 7.4), 2 mM MgCl2 and 1 mM

EDTA. After incubation, the samples were filtered rapidly under vacuum through glass fiber filters (GF/B, Packard Instruments, Meriden, CT, USA) presoaked with 0.3% polyethylenimine (PEI) and rinsed several times with ice-cold 50 mM Tris-HCl using a 96-sample cell harvester (Unifilter, Packard Instruments). The filters were dried, then counted for radioactivity in a scintillation counter (Topcount, Packard Instruments) using a scintillation cocktail (Microscint-O, Packard Instruments). The results were expressed as the percentage inhibition of the control radioligand-specific binding. The compounds were tested at a single concentration of 10 µM in triplicate.

Tested targets were as follows:

GPCR: A2A (agonist radioligand), α1A (antagonist radioligand), α2A (antagonist radioligand), β1 (agonist radioligand), β2 (agonist radioligand), BK1 (antagonist and agonist radioligand), BK2 (antagonist and agonist radioligand), CB1 (antagonist and agonist radioligand), CB2 (antagonist and agonist radioligand), CCK1 (CCKA) (agonist radioligand), D1 (antagonist radioligand), D2 (antagonist and agonist radioligand), D3 (antagonist and agonist radioligand), ETA (agonist radioligand), H1(antagonist radioligand), H2 (antagonist radioligand), M1 (antagonist radioligand), M2 (antagonist and agonist radioligand), M3 (antagonist radioligand), NK1(agonist radioligand), δ(DOP) (agonist radioligand), κ(KOP) (agonist radioligand), µ(MOP) (agonist radioligand), ORL1 (agonist radioligand), 5-HT1A (agonist radioligand), 5-HT1B (antagonist radioligand), 5-HT2A (agonist radioligand), 5-HT2B (agonist radioligand), V1a (agonist radioligand). Transporters: 5-HT transporter (antagonist radioligand), dopamine transporter (antagonist radioligand), norepinephrine transporter (antagonist radioligand). Ion Channels: 5-HT3 (antagonist radioligand), BZD (central) (agonist radioligand), NMDA (antagonist radioligand), N neuronal alpha4beta2 (agonist

radioligand), Ca2+channel (L.dihydropyridine site) (antagonist radioligand), Na+channel (site 2) (antagonist radioligand), KV (antagonist radioligand). Nuclear Receptors: AR (agonist radioligand), GR (agonist radioligand). Kinases and other non-kinase enzymes: CTK Lck kinase, acetylcholinesterase, PDE3A, PDE4D2, MAO-A (antagonist radioligand).

Finally, DF2726A was tested on TRPM8, TRPV1, TRPV4, TRPA1 and Nav1.7 ion channels in agonist and antagonist mode.

TRPM8-, TRPA1-, TRPV1-, TRPV4- and Nav1.7-expressing HEK-293 cells were analyzed in order to study the response to the compounds using a Ca2+ mobilization-dependent fluorescence signal in 384 MTP format. Cells were seeded at 10,000 cells per well in 384 MTP in complete medium (25 µl well-1). Twenty-four hours after seeding, the medium was removed and cells were loaded with 20 µL/well of the Fluo-8 NW dye solution. The dye-loaded cell plates were incubated for 1 h at RT. Test compounds at 3X-concentration in 1.5% DMSO Tyrode’s buffer were added to the wells of an assay plate, in 10 µL volume (for a final DMSO concentration of 0.5%) and read by the FLIPRTETRA plate. The kinetic response was monitored by the instrument over a period of 3 mi (180 seconds). A second injection of 10 µL well-1 of reference agonists (Capsaicin, GSK1016790A, Isothiocyanate and Veratridine for TRPA1, TRPV1, TRPV4 and Nav1.7, respectevely) at 4X-concentration in assay buffer (EC80) was added by the FLIPRTETRA. The signal of the emitted fluorescence was recorded for an additional 3 min.

DF2726A was tested at 8 concentrations in quadruplicate (30 µM was the highest tested concentration) to determinate the IC50 towards the panel of ion channels. The compound curve

fitting profile on each dose-response was performed with the Condoseo module of Genedata Screener 13.0.5.

DF2726A was tested on TRPM8, TRPV1, TRPV4, TRPA1 and Nav1.7 ion channels in agonist and antagonist mode.

Protein binding in human, dog and rat plasma

Stock solutions of DF2726A and the reference standards (Diclofenac and Antipyrine) were prepared in DMSO at a final concentration of 2 mM. Next, 1.5 µL of compound were spiked in 298.5 µL of plasma to obtain a final concentration of 10 µM. Human, dog and rat plasma (200 µL) in triplicate for the compound were dialyzed in the Rapid Equilibrium Dialysis (RED) inserts (ThermoFisher Scientific) against 350 µL of PBS in the RED plate for 4 h at 37°C, under agitation. At the end of the incubation, 50 µL of plasma were added to 50 µL of blank PBS buffer and 300 µL of acetonitrile (with 2.5 ng/mL of Verapamil as internal standard).

Similarly, 50 µL of incubated PBS were added to 50 µL of blank plasma and 300 µL of acetonitrile with Verapamil. Samples were centrifuged at 12000 x g for 5 min and surnatants were transferred to vials for LC-MS/MS analysis.

COX1-2 assay

DF2726A was tested to evaluate the activity towards human COX1 and COX2 by measuring the formation of PGE2 from arachidonic acid using a recombinant enzyme isolated from transfected Sf-9 cells.

The test compound, reference compound or water (control) were pre-incubated for 20 min at RT with the enzyme (≈ 5 µg for COX1 assay and ≈ 0.2 µg for COX2 assay) in a buffer containing 90

mM Tris-HCl (pH 8.0), 1.98 mM phenol and 1.02 µM hematine. Thereafter, the reaction was initiated by adding 4 µM (COX1) or 2 µM (COX2) of arachidonic acid and the mixture was incubated for 5 min at RT. For basal control measurements, arachidonic acid was omitted from the reaction mixture. Following incubation, the reaction was stopped by the addition of 1 M HCl then 1 M Tris/HCl (pH 8.0) followed by cooling to 4°C.

The fluorescence acceptor (d2 labeled PGE2) and the fluorescence donor (anti-PGE2 antibody labeled with europium Cryptate) were then added. After 120 min, the fluorescence transfer corresponding to the amount of residual PGE2 was measured at λex=337 nm, λem=620 nm and λem=665 nm using a microplate reader (Envision, Perkin Elmer). The enzyme activity was determined by dividing the signal measured at 665 nm by that measured at 620 nm (ratio). The results were expressed as a percentage inhibition of the control enzyme activity. The standard inhibitory reference compound was Diclofenac, which was tested in each experiment at several concentrations to obtain an inhibition curve from which its IC50 value was calculated. DF2726A was tested at a single concentration of 50 µM in triplicate.

hERG inhibition

The human ether-a-go-go related gene (hERG) inhibition assay was performed by Eurofins Panlabs Inc, USA. DF2726A was tested to assess the potential interaction on hERG channel stably transfected in Chinese hamster ovary (CHO-K1) cell line. Single cell ionic currents were measured in the automated patch clamp configuration at room temperature by using QPatch 16 instrument. The compound was tested at 5 increasing concentrations (0.01, 0.05, 0.1, 0.3 and 1 mM) in triplicate to obtain the IC50 values.

AMES assay

DF2726A was tested to investigate the potential gene mutation induction by means of the Salmonella Typhimurium reverse mutation assay in TA98, TA100, TA1535 and TA1537 strains. The assay was performed with and without liver microsomal activation by using rat liver S9 fraction. The compound was tested at the following concentrations: 100 µM, 50 µM, 10 µM, 5 µM (n = 12). The bacterial plates were incubated with the test compound for 96 hours, after which bacterial growth was measured spectrophotometrically using a pH indicator that changes color in response to the acidification of the media due to bacterial growth. To prevent false negatives due to bactericidal or bacteriostatic effects, a bacterial cytotoxicity assay was conducted in parallel with the Ames fluctuation assay (8 concentrations with 100 µM as the highest concentration and n = 3). Four reference compounds (quercetin, streptozotocin, aminoanthracene and aminoacridine) were included in all assays.

Pharmacokinetics studies in the rat

DF2726A was administered to male Sprague Dawley rats (Charles River Srl. Calco Italy, 6-8 weeks old, n = 4/groups) intravenously (5 mg/kg, Dulbecco's phosphate buffered saline) and orally (5 mg/kg Dulbecco's phosphate buffered saline).

On day of treatment, an exact amount of DF2726A was dissolved in the appropriate volume of vehicle to obtain a final concentration of 2.0 mg/mL (IV) and 1 mg/mL (OS). Both of the formulations were filtered at 0.45 µm filter before use in animals.

After the administration of DF2726A, a 0.15-0.20 mL of blood was collected via jugular vein catheters with zero-dead volume disposable syringes (1 mL iv Insulin syringes with attached 25G

5/8 inch needles; Chemil s.r.l., Padova, Italy) at 0.08, 0.5, 1, 3, 6, 12, 24, 36 and 48 h for i.v. dosing and at 0.25, 0.5, 1, 3, 6, 12, 24, 36 and 48 h for oral dosing. After each blood withdrawal, the catheters were washed with 0.15-0.2 mL of sterile physiological saline and filled with 0.03 mL of heparin saline solution (0.25 U.I/mL). The heparinized blood was immediately centrifuged (8000 RPM for 5 min at approximately 4°C) and the resultant plasma transferred into vials and stored at -20°C until analysis. DF2726A concentrations were quantified by HLPC-MS/MS using a chromatographic method with a lower limit of quantitation of 1 ng·mL-1. Non-compartmental analysis (Gibaldi and Perrier, 1982) were used to calculate the pharmacokinetic parameters using a MKModel by Nick Holford software Version 4, (Biosoft, Ferguson, MO 63135, USA).

Drug Administration

DF2726A was administered at a dose of 30 mg/2ml/kg/os for 14 consecutive days starting 3 days before paclitaxel administration and continuing for 11 days after the first administration of paclitaxel.

Induction of neuropathy by paclitaxel

Rats received 4 once daily intraperitoneal (i.p.) injections of paclitaxel (Tocris, Italy) (2 mg/kg/day i.p.; cumulative dose of 8 mg/kg i.p.) or vehicle (saline, 1ml/kg/day i.p.), administered on alternate days (days 0, 2, 4, and 6) as described [4]. Behavioral testing was performed prior to paclitaxel/vehicle administration (day-1) in order to determine the basal values of the mechanical

and cold nociceptive thresholds, and again on 5, 7, 10 and 14 days following paclitaxel/vehicle injection as described [5].

Mechanical allodynia

To assess for changes in sensation or in the development of mechanical allodynia, sensitivity to tactile stimulation was measured using the Dynamic Plantar Aesthesiometer (DPA, Ugo Basile, Italy). Animals were placed in a chamber with a mesh metal floor covered by a plastic dome that enabled the animal to walk freely, but not to jump. The mechanical stimulus was then delivered in the mid-plantar skin of the hind paw. The cut-off was fixed at 50 g. Testing was performed on both paws before (day -1) and then on 5, 7, 10 and 14 days after paclitaxel administration.

Cold allodynia

Cold sensitivity was measured as the number of foot withdrawal responses after application of acetone to the dorsal surface of the paw [6]. A drop of acetone was applied to the dorsal surface of paws with a syringe connected to a thin polyethylene tube while the rats were standing on a metal mesh. A brisk foot withdrawal response, after the spread of acetone over the dorsal surface of the paw, was considered as a sign of cold allodynia. Data represent mean of 3 measurements performed at an interval of approximately 5 min.

Cold responses were measured on both paws before (day -1) and then on 5, 7, 10 and 14 days after

Cell treatments

F11 hybridoma cells were treated for 24 hours with DF2726A (Dompé farmaceutici S.p.A.; 10 µM final concentration), paclitaxel (Sigma-Aldrich; 10 nM final concentration) and the combination of the two molecules.

Paclitaxel stock solution (10 mM) was prepared by dissolving the powder in DMSO, and aliquots were stored at -20°C.

References

1. Di Cioccio, V. *et al.* Key role of proline-rich tyrosine kinase 2 in interleukin-8 (CXCL8/IL-8)-mediated human neutrophil chemotaxis. *Immunology* **111**, 407-415, doi:10.1111/j.1365-2567.2004.01822.x (2004).
2. Bertini, R. *et al.* Noncompetitive allosteric inhibitors of the inflammatory chemokine receptors CXCR1 and CXCR2: prevention of reperfusion injury. *Proc Natl Acad Sci U S A* **101**, 11791-11796, doi:10.1073/pnas.0402090101 (2004).
3. Bowes, J. *et al.* Reducing safety-related drug attrition: the use of in vitro pharmacological profiling. *Nat Rev Drug Discov* **11**, 909-922, doi:10.1038/nrd3845 (2012).
4. Polomano, R. C., Mannes, A. J., Clark, U. S. & Bennett, G. J. A painful peripheral neuropathy in the rat produced by the chemotherapeutic drug, paclitaxel. *Pain* **94**, 293-304 (2001).
5. Brandolini, L. *et al.* CXCR1/2 pathways in paclitaxel-induced neuropathic pain. *Oncotarget* **8**, 23188-23201, doi:10.18632/oncotarget.15533 (2017).
6. Choi, Y., Yoon, Y. W., Na, H. S., Kim, S. H. & Chung, J. M. Behavioral signs of ongoing pain and cold allodynia in a rat model of neuropathic pain. *Pain* **59**, 369-376 (1994).

Supplementary figures legends

Supplementary Fig. 1.

In control animals administered with paclitaxel vehicle i.p. (CTR, black dots) the paw withdrawal responses remained unchanged during the whole experimental period. Rats treated with paclitaxel (Pac) + DF2726A vehicle (Pac+Saline, grey squares), showed marked changes in paw withdrawal responses. Administration of DF2726A (Pac+DF, light grey triangles) was able to reduce in a significant way, paclitaxel-evoked cold and mechanical allodynia. ***$P < 0.001$, **$P < 0.01$ and *$P < 0.05$ vs respective CTR group; ###$P < 0.001$, ##$P < 0.01$ and #$P < 0.05$ vs the respective Pac+Saline group. 2-way repeated-measures ANOVA and Bonferroni test. Data are expressed as mean ± SEM; n 10 per group. ANOVA, analysis of variance.

Supplementary Fig. 2: Effect of DF2726A, oxaliplatin and paclitaxel treatment on neuronal cell viability. Cell viability assay of F11 cells after treatment at different concentrations of DF2726A (DF; 1-20 μM), oxaliplatin (OXA, 5-60 μM), paclitaxel (PAC, 5-20 nM) and the combination of 20 μM oxaliplatin+DF2726A (Oxa+DF) and paclitaxel+DF2726A (PAC+DF) at 24 h. Data are means ± SEM of 5 different experiments. *$P < 0.05$ vs control; +$P < 0.05$ vs Oxa, .

Supplementary Fig. 3: Effect of DF2726A treatment on acetylated α-tubulin in paclitaxel-treated neurons. A) Representative images of immunofluorescence analyses of acetylated α-tubulin (green) in control (CTR), paclitaxel (Pac)-, DF2726A- and paclitaxel+DF2726A (Pac+DF)-treated neurons. Nuclei are stained with DAPI (blue). Bar = 75 μm. B) Representative blots probed for acetylated α-tubulin (right panel) and quantification (left panel) of acetylated α-tubulin in the same treatment conditions. Data are means ± SEM of 3 different experiments. **$P < 0.005$ versus control; +$P < 0.05$, versus OXA

Supplementary Fig. 4: Effect of DF2726A treatment on paclitaxel-induced neurotoxic pathways. Representative blots and relative quantitations of p-FAK, p-JAK2, p-STAT3, PI3K and p-cortactin

in control (CTR), paclitaxel (PAC)-, DF2726A (DF)- and paclitaxel+DF2726A (PAC+DF)-treated neurons. Data are means ± SEM of 3 different experiments. **$P < 0.005$, *$P < 0.05$ vs control; +$P < 0.05$, ++$P < 0.005$, +++$P < 0.0005$ vs Oxa

Supplementary figures

Fig. 1

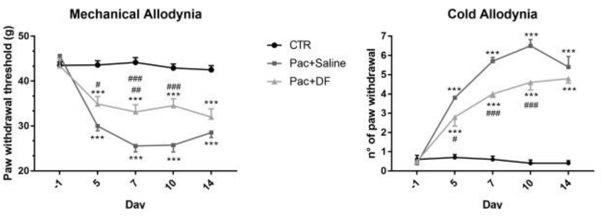

Fig. 2

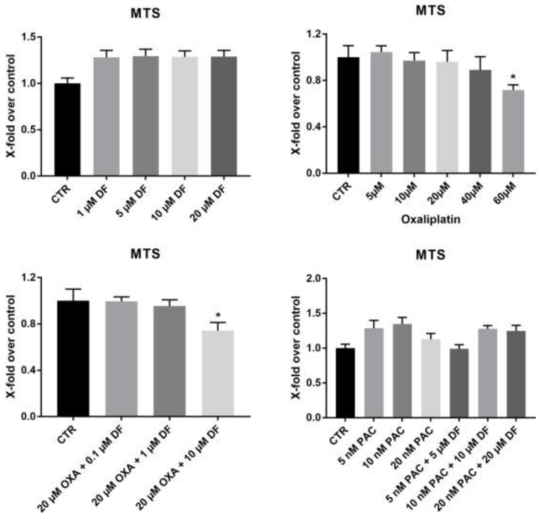

Fig. 3

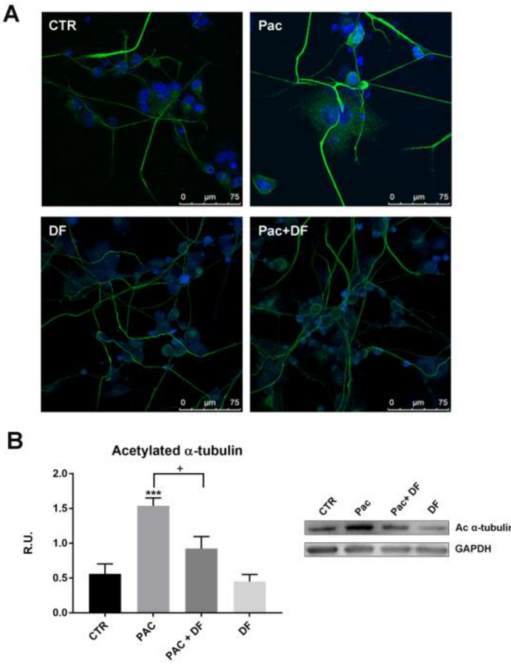

Fig. 4

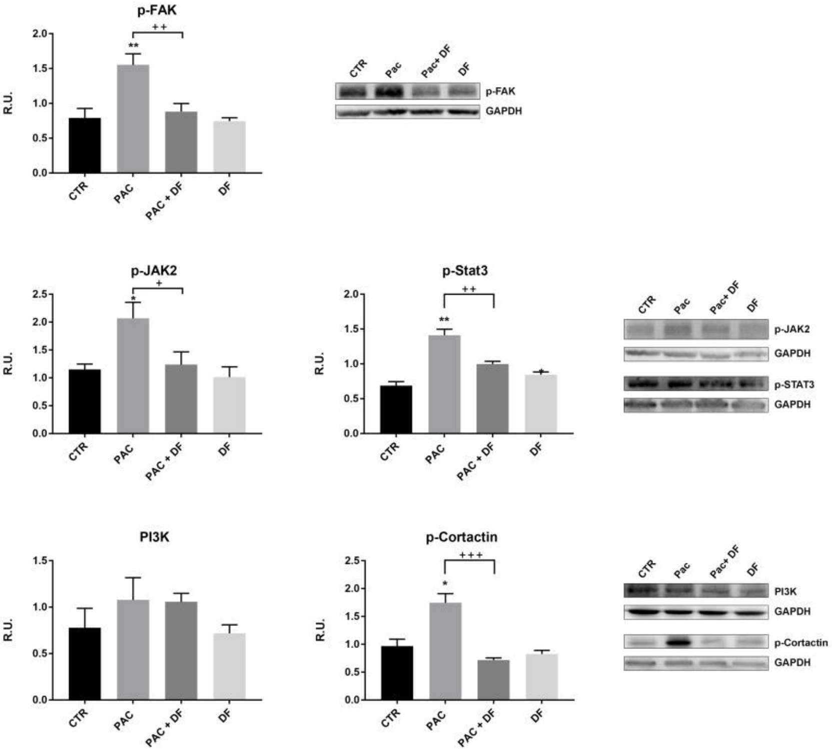

Supplement: Supplementary file 1 — supplementary informations [file 41598_2019_48231_MOESM1_ESM.pdf]
